# Programmed Death-Ligand 1 as a Regulator of Tumor Progression and Metastasis

**DOI:** 10.3390/ijms22105383

**Published:** 2021-05-20

**Authors:** Ioannis A. Vathiotis, Georgia Gomatou, Dimitrios J. Stravopodis, Nikolaos Syrigos

**Affiliations:** 1 Department of Medicine, School of Medicine, National and Kapodistrian University of Athens, 15772 Athens, Greece; georgiagomatou@gmail.com (G.G.); nksyrigos@gmail.com (N.S.); 2 Department of Pathology, Yale University School of Medicine, New Haven, CT 06510, USA; 3 Department of Biology, School of Medicine, National and Kapodistrian University of Athens, 15772 Athens, Greece; dstravop@biol.uoa.gr

**Keywords:** PD-1, PD-L1, cancer, progression, metastasis

## Abstract

Programmed cell death protein 1 (PD-1)/programmed death-ligand 1 (PD-L1) immune checkpoint has long been implicated in modeling antitumor immunity; PD-1/PD-L1 axis inhibitors exert their antitumor effects by relieving PD-L1-mediated suppression on tumor-infiltrating T lymphocytes. However, recent studies have unveiled a distinct, tumor-intrinsic, potential role for PD-L1. In this review, we focus on tumor-intrinsic PD-L1 signaling and delve into preclinical evidence linking PD-L1 protein expression with features of epithelial-to-mesenchymal transition program, cancer stemness and known oncogenic pathways. We further summarize data from studies supporting the prognostic significance of PD-L1 in different tumor types. We show that PD-L1 may indeed have oncogenic potential and act as a regulator of tumor progression and metastasis.

## 1. Introduction

Manipulation of the immune system to control tumor growth can be traced back to 1891, when William Coley used live bacteria as an immune stimulant to treat cancer, founding the field of tumor immunology [1]. The immune system is governed by the crosstalk among different immune checkpoints that are normally responsible for maintaining the immune response within a desired physiologic range and protecting the host from autoimmunity; tumors employ immune checkpoints to escape immune recognition and evade immune attack [2,3,4]. As such, programmed cell death protein 1 (PD-1)/programmed death-ligand 1 (PD-L1) regulatory system transmits inhibitory signals, forming a negative feedback loop to constrict local T cell responses and preserve peripheral tolerance. PD-1 is mainly found on activated T cells [5]. Upon interaction with PD-L1, PD-1 recruits tyrosine phosphatase Src homology 2 domain-containing phosphatases 1/2 (SHP1/2) that dephosphorylate proximal signaling elements and directly attenuate TCR signaling [6,7,8].

PD-L1 (also known as CD274 or B7H1) is principally expressed on tumor and antigen-presenting cells (APCs) [6,7]. Typically, PD-L1 expression is induced by IFNγ and, acting through downstream regulators including JAK and STAT, mediates binding of the IRF1 transcription factor to the promoter of the *PD-L1* gene [9]. Besides this, oncogenic pathways, including RAS/RAF/MEK/ERK and PI3K/Akt/mTOR, have been shown to induce the expression of PD-L1 through activation of c-JUN that, as a component of AP-1 transcription factor, binds to the enhancer element on the *PD-L1* gene and augments the transcription signal in tumor cells [10]. Assessment of PD-L1 expression by immunohistochemistry (IHC) is a predictive biomarker of response to anti-PD-1/PD-L1 treatment; across different tumor types, patients with PD-L1-negative tumors responded to PD-1/PD-L1 axis inhibitors in 0–17% of cases, while those with PD-L1-positive tumors responded in 44–100% of cases [11].

However, tumor-intrinsic PD-L1 signaling has been less well defined [12,13]. Here we review the mechanisms by which PD-L1 regulates tumor progression and metastasis, with a focus on PD-L1 expression on cancer stem-like cells, exosomal PD-L1, integrin signaling related to PD-L1, PD-L1 stabilization and epigenetic regulation of PD-L1 expression. Then, we summarize clinical data on the prognostic significance of PD-L1 expression in patients with malignancy.

## 2. PD-L1 Expression on Cancer Stem-Like Cells (CSCs)

Although CSCs, also known as tumor-initiating cells, represent a small minority of tumor cells, they share numerous critical properties, such as the ability of self-renewal, differentiation and dedifferentiation, rendering them pivotal for tumor initiation, progression, metastatic dissemination and recurrence [14,15]. CSCs are immune-privileged; they are able to escape immune recognition. As indicated by Schatton et al., the immunogenic tumor-associated melanoma antigen recognized by T cells 1 (MART-1) is expressed on differentiated rather than malignant melanoma initiating cells (MMICs), rendering the latter poor targets for immunosurveillance [16,17]. Similar results were reported in preclinical breast cancer models [18]. In addition, CSCs are poor antigen-presenters, as they express high levels of immunosuppressive major histocompatibility complex (MHC) class Ib, low levels of immunostimulatory MHC class Ia and no MHC class II molecules [19]. Moreover, CSCs may actively suppress immune responses; MMICs have been shown to preferentially inhibit IL-2 production and induce regulatory T cells (Tregs) [16,19,20]. Interestingly, through the concept of immunoediting, the immune system is also capable of prompting tumor cells to undergo epithelial-to-mesenchymal transition (EMT) and acquire stem cell features, giving rise to a more aggressive phenotype [21].

**Figure 1:**
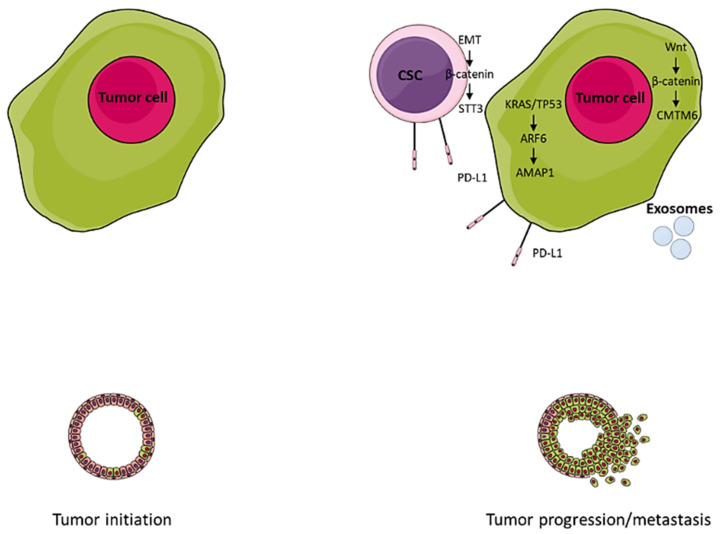
Tumor intrinsic programmed death-ligand 1 signaling mediates tumor progression and metastasis. CSC, cancer stem-like cell; EMT, epithelial-to-mesenchymal transition; PD-L1, programmed death-ligand 1; CMTM6, chemokine-like factor-like MARVEL transmembrane domain-containing family member 6.

Whereas both the canonical and noncanonical Notch pathways have been implicated in the generation of CSCs, only the noncanonical Notch pathway was found to be involved in PD-L1 upregulation in colorectal CSCs [22]. Hsu et al. proposed a CSC-specific mechanism for the induction of PD-L1 expression via the EMT/β-catenin/STT3 signaling axis [23] (Figure 1). In this model, either STT3 isoform, STT3A or STT3B, was sufficient and required for PD-L1 N-glycosylation and stabilization (Table 1). Moreover, reversal of EMT by etoposide caused PD-L1 downregulation in both CSC and non-CSC populations, advocating combinatorial approaches with PD-1/PD-L1 axis inhibitors.

Recent studies have suggested that PD-L1 expression may contribute to CSC immune evasion. A preclinical model in head and neck squamous cell carcinoma (HNSCC) demonstrated that CD44+ cells have significantly increased PD-L1 expression, both constitutive and inducible, in comparison with their CD44- counterparts [24]. In addition, PD-L1 expression was both biologically and clinically relevant since PD-1 blockade was able to reverse to a certain extent the decreased immunogenicity of CD44+ HNSCC cells. Using CD133 and EpCAM to mark CSCs, Raniszewska et al. established the presence of PD-L1 on presumed CSCs from lymph node metastases in endobronchial ultrasound-guided transbronchial needle aspiration (EBUS-TBNA) samples from patients with non-small-cell lung cancer (NSCLC) by flow cytometry [25]. PD-L1 expression on CSCs was increased in comparison with mature tumor cells, serving as an indication of loss of such molecules during differentiation [26]. Moreover, PD-L1 expression on CSCs was associated with an altered immune cell infiltrate; PD-L1 overexpression was correlated with increased infiltration of Tregs, PD-1+/CD4+ T cells and Tim3+/CD4+ T cells [27]. Thus, upregulation of PD-L1 on CSCs, but not tumor cells, may be responsible for the induction of CD4+ T cell anergy. Finally, both PD-L1 upregulation and CD4+ T cell phenotypic change contributed to an aggressive behavior and resistance to conventional therapies, including chemotherapy and radiotherapy, conferring a poor prognosis overall. Similarly, PD-L1 expression on pancreatic CD44+/CD133+ CSCs led to poor clinical outcomes, namely disease recurrence [28]. Induction of PD-L1 by tumor-associated mesenchymal stem cells enhanced stemness potential, tumorigenicity and chemoresistance of tumor cells in a preclinical gastric cancer model [29]. Finally, surface expression of PD-L1 was linked with signatures of immune evasion as well as increased stemness of epithelial bladder cancer cells [30].

## 3. Exosomal PD-L1

Exosomes are lipid bilayer extracellular vesicles, with a size of 40–150 nm, that are naturally produced and released by all cell types [31]. Exosomes are highly heterogeneous. They mirror the orientation of the lipid bilayer and reflect the phenotypic state of the cell of origin. Carrying nucleic acids or proteins, they are biologically active and play a central role in intercellular communication. While part of the exosomal protein cargo reflects the common exosome biogenesis pathway, exosomal proteins may be enriched with respect to the type and current state of the parent cell. Exosomes are upregulated in disease states; the blood of cancer patients is estimated to contain about 4000 trillion exosomes [32,33]. Exosomes have been shown to promote tumorigenesis, tumor progression and metastasis and facilitate remodeling of the extracellular matrix (ECM), tumor angiogenesis and activation of cancer-associated fibroblasts. Additionally, they have been implicated in immune regulation with both protumor and antitumor properties [34].

Tumor-derived exosomes may contain PD-L1 both on the surface and within; proteins involved in packaging and secretion of exosomal PD-L1 include endosomal sorting complex required for transport (ESCRTs), neutral sphingomyelinase 2 (nSMase2) and Rab27a [35]. Upregulated by IFNγ, exosomal PD-L1 can recapitulate the effects of cell-surface PD-L1 [36]. PD-L1 on exosomes can directly engage PD-1+ T cells in the tumor microenvironment (TME), leading to suppression of T cell responses and CD8+ T cell exhaustion [37]. PD-L1-containing exosomes have been involved in tumor progression in preclinical malignant glioma models; exosomal PD-L1 secreted by glioblastoma stem-like cells inhibited the activation of CD4+ and CD8+ immune effector cells [38]. Exosomes isolated from non-small-cell lung cancer (NSCLC) patients reduced IFNγ and IL-2 production and induced apoptosis in CD8+ T cells, favoring tumor growth [39]. The exosome may also serve as a vehicle, delivering PD-L1 to different cell types within the TME, and as such model antitumor immune response [40]. Current evidence has confirmed the presence of PD-L1 on exosomes secreted by osteosarcoma cells [41]. Patients with osteosarcoma had higher levels of exosomal PD-L1 than healthy donors. Furthermore, the presence of pulmonary metastases was associated with relatively increased levels of exosomal PD-L1 as compared to the absence of metastases. PD-L1-containing exosomes were implicated in immune evasion and tumorigenesis, whereas coexpression of PD-L1 and N-cadherin on exosomes was predictive of metastatic dissemination.

Of note, eradication of exosomal PD-L1 impeded tumor growth even in tumor models resistant to PD-1/PD-L1 axis inhibitors, while systemic introduction of exosomal PD-L1 rescued tumor growth in tumors unable to produce their own [42]. Recently, the levels of exosomal but not soluble PD-L1 were associated with evidence of advanced disease as well as disease activity in patients with HNSCC and NSCLC [43,44]. Interestingly, exosomal PD-L1 levels showed no correlation with PD-L1 tumor proportion score by IHC [44]. Plasma levels of exosomal PD-L1 have been negatively correlated with response rate to anti-PD-1/PD-L1 therapy as well [36]. Thus, elimination of circulating exosomes, employing either pharmacological approaches or extracorporeal hemofiltration, has emerged as a novel therapeutic strategy to inhibit metastatic dissemination and improve the efficacy of anticancer therapy.

## 4. Integrin Signaling

Integrins are a family of 24 unique, bidirectional signaling αβ heterodimers that mediate cell adhesion to the extracellular matrix, as well as cell-to-cell adhesions [45]. Integrin activation triggers the recruitment of the so-called adhesome, which represents a complex node of signaling, scaffolding and cytoskeletal proteins engaging directly or indirectly with integrin cytoplasmic tails. This formation represents highly dynamic machinery responsible for regulating aspects of cell fate such as survival, migration, polarity and differentiation [46]. Altered integrin expression patterns have been associated with different types of cancer [46,47,48]. In addition, integrins have been implicated in leukocyte adhesion and the regulation of immune responses.

Several studies have linked integrin signaling with PD-L1 expression in human solid tumors. Classic oncogenic drivers of pancreatic ductal adenocarcinoma have been shown to activate β1 integrin (ITGB1) signaling pathways to promote immune evasion and tumor progression, through PD-L1 upregulation [49]. Specifically, KRAS promoted *ARF6* and *AMAP1* mRNA translation, while TP53 facilitated ARF6 activation by PDGFR and MVP. Altogether, the ARF6/AMAP1 axis promoted PD-L1 dynamics, augmenting PD-L1 recycling and cell surface expression, which ultimately led to immune evasion in vivo. These results were replicated in human pancreatic ductal adenocarcinoma, where AMAP1 expression was correlated with PD-L1 expression and fibrosis, ultimately leading to poor patient outcomes [50]. Furthermore, β3 integrin (ITGB3) signaling led to both inducible, through IFNγ, and constitutive upregulation of PD-L1 expression [51]. The implantation of β3-integrin-depleted tumor cells led to immune “hot” tumors, with decreased PD-L1 expression, increased local IFNγ production and CD8+ T cell infiltration, diminishing tumor growth. Depletion of β3-integrin elicited an abscopal immunotherapeutic effect measured as protection from the challenge tumor and durable splenocyte and serum reactivity to tumor antigens, providing the rationale for combinatorial immunotherapeutic approaches with PD-1/PD-L1 axis inhibitors. In cervical cancer, PD-L1 bound to β4 integrin (ITGB4) and directly affected genes associated with EMT and glucose metabolism through the ITGB4/SNAI1/SIRT3 signaling axis, highlighting a tumor-intrinsic role for PD-L1 [52]. Bioluminescence imaging analysis of cervical xenograft tumors in mice revealed that PD-L1 overexpression augmented tumor glucose uptake. High expression of PD-L1 and ITGB4 in human cervical carcinomas was significantly associated with lymph node metastasis and poor prognosis. Finally, Cao et al. presented evidence of retinoic acid related orphan receptor C (RORC) binding to the promoter region and negatively regulating PD-L1 expression in bladder cancer [53]. PD-L1 was shown to directly interact with β6 integrin (ITGB6), enhance tumor cell proliferation, alter glucose metabolism, and overall promote tumor progression.

## 5. PD-L1 Stabilization

Chemokine-like factor (CKLF)-like MARVEL transmembrane domain-containing family member 6 (CMTM6) is an omnipresent transmembrane protein that belongs to a family of eight MARVEL domain-containing proteins [54]. CMTM6 increases PD-L1 protein pool without affecting PD-L1 transcript levels; present on the cell surface, CMTM6 specifically reduces PD-L1 protein ubiquitination and lysosomal degradation, thereby stabilizing PD-L1 and increasing its protein half-life. Indeed, CMTM6 represents a master regulator of PD-L1 cell surface expression across various cancer types [55]. Presence of CMTM6 augmented T cell inhibitory capacity of PD-L1-expressing tumor cells, whereas CMTM6 depletion mitigated the attenuation of tumor-specific T cell activity both in vitro and in vivo. Interestingly, INFγ was not correlated with the CMTM6 pathway. Recently, Hu et al. identified Hu-Antigen R (HuR) as a regulator of CMTM6; HuR directly engaged AU-rich elements in the 3′ untranslated region and stabilized CMTM6 mRNA [56]. Importantly, CMTM6-carrying exosomes induced M2 macrophage polarization by activating the ERK1/2 signaling pathway in a preclinical oral squamous cell carcinoma model [57].

CMTM6 has been shown to refine the prognostic value of PD-L1 in solid tumors [58]. However, a pan-cancer analysis revealed that the prognostic pertinence of CMTM6 might be tumor-specific [59]. CMTM6 overexpression by IHC predicted poor outcomes in patients with HNSCC [60]. Presence of CMTM6 led to the activation of the Wnt/β-catenin signaling pathway, which has been implicated in tumorigenesis, EMT, cancer stemness and T cell dysfunction [61]. Contrariwise, CMTM6 silencing downregulated PD-L1 and ultimately led to delayed tumor growth and increased T cell infiltration. Another study in triple-negative breast cancer revealed that the expression of CMTM6 was correlated with that of PD-L1 [62]. Furthermore, higher CMTM6 expression was an independent risk factor for shorter progression-free survival (PFS). Similarly, coexpression of CMTM6 and PD-L1 had a negative impact on both disease-free survival (DFS) and overall survival (OS) of patients with hepatocellular carcinoma [63]. However, coexpression of CMTM6 in tumor cells and PD-L1 in stromal cells was associated with longer OS in patients with colorectal cancer [64]. Finally, CMTM6 expression in either tumor or stroma was not correlated with prognosis in patients with NSCLC [65].

Epithelial cell adhesion molecule (EpCAM, CD326) is a transmembrane glycoprotein that represents the most commonly expressed epithelial tumor-derived antigen [66,67]. Its extracellular domain (EpEX) has been shown to activate epidermal growth factor receptor (EGFR) and, through ERK1/2 signaling, promote tumor cell proliferation, migration, and invasion. Furthermore, the previously described axis mediates regulated intramembrane proteolysis of EpCAM, sequentially by TNFa-converting enzyme (TACE) and γ-secretase, and induces nuclear accumulation of its intracellular domain (EpICD), which has been shown to possess tumorigenic potential, through the upregulation of reprogramming genes and EMT [68,69]. Overexpression of EpCAM has been identified as a negative prognostic factor in various cancer types [66].

In addition to driving tumor progression, EGFR activation is closely related to PD-L1 signaling. EGFR-activating mutations have been correlated with overexpression of PD-L1 in preclinical EGFR-driven lung cancer models, linking EGFR signaling with immune evasion [70]. Both the MAPK and p-ERK1/2/p-c-Jun signaling pathways upregulated *PD-L1* mRNA [71]. EGFR tyrosine kinase inhibitors relieved PD-L1-mediated T cell inhibition and upregulated IFNγ production. EGFR ligands promoted PD-L1 glycosylation to stabilize PD-L1 and prevent glycogen synthase kinase 3β (GSK3β)-mediated proteasomal degradation by β-TrCP [72]. EpCAM and soluble EpEX bound directly to EGFR through the EGF-like domain I and induced EGFR signaling [73]. Additionally, EpCAM drove MAPK-mediated PD-L1 glycosylation and stabilization, ultimately leading to immune escape through the EpEX/EGFR/ERK signaling axis. Finally, EpCAM blockade not only promoted apoptosis in tumor cells but also decreased PD-L1 protein levels to enhance the cytotoxic activity of CD8+ T cells.

## 6. Genetic and Epigenetic Regulation of PD-L1 Expression

PD-L1 is encoded by the *PD-L1* gene, located in chromosome 9p24.1, in close proximity to *PDCD1LG2* (programmed cell death 1 ligand 2). *PD-L1* gene amplification, assessed by fluorescence in situ hybridization (FISH), has been documented in a subset of patients with oral squamous cell carcinoma [74]. Interestingly, *PD-L1* gene amplification was concordant in primary tumors and associated nodal metastases. Both *PD-L1* and *PD-L2* copy numbers were found to be increased in patients with NSCLC; *PD-L1* copy number gains were independently associated with PD-L1 expression [75]. Again, *PD-L1* copy number status showed high consistency in primary tumors and corresponding lymph node metastases. Moreover, *PD-L1* gene amplification and increased PD-L1 protein expression were associated with significantly shorter overall survival in patients with NSCLC. *PD-L1* gene amplification has also been documented in gastric and triple-negative breast cancer [76,77].

Preliminary studies have demonstrated an inverse correlation between *PD-L1* promoter methylation (m*PD-L1*) and *PD-L1* mRNA expression [78,79]. Furthermore, m*PD-L1* was an independent prognostic factor in patients with colorectal cancer.

Causing loss of cell-to-cell adhesions and increasing cell motility and invasiveness in the surrounding stroma, EMT developmental program prompts metastatic dissemination of cancer cells [80]. Occurring in parallel with EMT, changes in the TME lead to the suppression of antitumor immunity. MicroRNAs (miRNAs) are short noncoding RNAs that could negatively regulate gene expression by binding to their 3′ untranslated regions (UTRs). They play a central role in the cell cycle and control multiple cellular properties, including cell proliferation, apoptosis, migration, and invasion; dysregulation of miRNAs has been linked with tumorigenesis. Recently, the EMT regulatory miR-200/ZEB1 axis was shown to attune PD-L1 expression in tumor cells [81]. miR-200/ZEB1 axis represents a double-negative feedback loop that governs a reversible switch between epithelial and mesenchymal states [82]. In addition, it acts as an upstream regulator of PD-L1, contributing to its immunosuppressive effects in the primary tumor. PD-L1 blockade significantly increased CD8+ T cell infiltration, reversed T cell exhaustion and reduced tumor burden and metastases in mesenchymal but not epithelial tumors, whereas PD-L1 reconstitution reversed the phenotype of epithelial tumors. Even in the absence of IFNγ, activation of the miR-200/ZEB1 axis resulted in induction of T cell exhaustion in mesenchymal tumor cells, evading immune recognition and attack. However, IFNγ stimulation and miR-200 repression seem to be intertwined, providing an example of how different modes of PD-L1 regulation may converge at the cellular level. Another miRNA, miR-502-5p, has been shown to act as a tumor suppressor; miR-502-5p was downregulated both in gastric cancer cells and human gastric cancer tissues in comparison with adjacent normal tissues, whereas overexpression of miR-502-5p impeded tumor growth, migration and metastasis in vitro and in vivo [83]. miR-502-5p repressed *PD-L1* mRNA expression both directly by binding to its 3′ UTR and indirectly by suppressing CD40 and STAT3 expression.

Furthermore, the long noncoding RNA (lncRNA) PSMB8-AS1 acted as a miRNA sponge to inhibit the expression of miRNA-382–3p and upregulate PD-L1 via the miR-382–3p/STAT1/PD-L1 axis [84]. Once again, PD-L1 was an effective regulator of pancreatic cancer, as PD-L1 knockdown could confine the tumorigenicity of pancreatic cancer cells. Lately, Mu et al. established a protumorigenic role for lncRNA hypoxia-inducible factor-1 alpha antisense RNA-2 (HIF1A-AS2) [85]. In this study, increased levels of HIF1A-AS2 were reported in gastric cancer tissues and cell lines. Moreover, HIF1A-AS2 promoted tumor cell proliferation and metastatic dissemination through the miR-429/PD-L1 axis; HIF1A-AS2 directly inhibited miR-429 to regulate PD-L1 expression and bolster tumor progression and metastasis.

Though not typically detected by standard methodologies that are used to profile human tumors (e.g., genomics, transcriptomics), translational control of the immune regulators, including PD-L1, facilitates immune evasion and promotes tumor progression and metastasis. In particular, although a potent upstream open reading frame embedded in the 5′ untranslated region of *PD-L1* mRNA represses its efficient translation, specific oncogenes, such as *KRAS* and *MYC*, were able to bypass this obstacle through eIF2a phosphorylation [86]. Cancer cells may use this pathway to more rapidly synthesize PD-L1 protein (compared with transcriptional control), in response to changes in the TME, enabling tumor progression and metastasis. Furthermore, structural variations disrupting its 3′ untranslated region have been shown to stabilize *PD-L1* mRNA and aid in immune evasion in multiple types of cancer [87].

## 7. Prognostic Implications

Lately, many studies have examined the heterogeneity in PD-L1 protein expression in patients with solid tumors. Several studies have provided preliminary indications of considerable intertumoral heterogeneity for PD-L1 expression in patients with NSCLC [88,89,90,91]. Hong et al. demonstrated that PD-L1 tumor proportion score (TPS) was significantly elevated in samples obtained from specific metastatic sites, including adrenal gland, liver and lymph node metastases, in comparison with primary lesions of NSCLC patients [92]. Temporal variability in PD-L1 protein expression has also been recorded, with substantial decreases in PD-L1 TPS in early-stage disease as well as after exposure to immunotherapy [92,93]. Moreover, different sampling sites of PD-L1 may carry different predictive values; PD-L1 biopsies obtained from lymph nodes were not associated with either response or survival upon treatment with immune checkpoint inhibitors [92]. Dave et al. assessed PD-1 and PD-L1 expression in oral epithelial dysplasia lesions that progressed to oral squamous cell carcinoma (OSCC), lesions that did not and OSCC lesions [94]. Notably, PD-L1 expression was significantly increased in both basal epithelial and immune cells of progressing rather than non-progressing oral epithelial dysplasia lesions. These results indicate that the PD-1/PD-L1 immune checkpoint facilitates malignant transformation of premalignant lesions; activation of the PD-1/PD-L1 interaction might occur well before malignant transformation. All the above represent corroborative evidence supporting the notion that PD-L1 might act as a driver of tumor progression.

Furthermore, several studies have highlighted the prognostic significance of PD-L1 in different tumor types. Increased PD-L1 expression was associated with shorter OS in patients with NSCLC [93,95]. In addition, PD-L1 expression was an independent risk factor of death in patients with mesothelioma [96]. Interestingly, two recent meta-analyses provided conflicting results regarding the association of PD-L1 positivity and the presence of lymph node metastases in patients with melanoma [97,98]. PD-L1 overexpression was not associated with OS in patients with melanoma; when restricted to metastatic melanoma, PD-L1 positivity was correlated with prolonged OS. In cutaneous squamous cell carcinoma, high RNA or protein PD-L1 expression in the primary tumor was associated with an increased risk of metastatic dissemination [99]. A recent meta-analysis of 55 studies showed that increased PD-L1 expression was overall a negative prognostic factor in patients with gynecological cancers; this was driven by a favorable OS for patients with ovarian cancer but a poor OS for patients with cervical cancer [100]. High PD-L1 expression was also associated with poor OS in patients with sarcoma [101]. Consistent with previous reports, Jiang et al. showed that PD-L1 positivity was associated with more aggressive disease features, including deeper tumor invasion and more nodal metastases, ultimately leading to significantly shorter OS in patients with esophageal squamous cell carcinoma treated with definitive therapy, such as curative esophagectomy or definitive (chemo)radiation [102,103,104].

Quantification of PD-L1 concentration in the serum (sPD-L1) has recently gained attention since it represents a quick, easy, and cost-effective solution for the assessment of PD-L1 expression in patients with malignancy. Plasma sPD-L1 was present in the supernatant of breast cancer cell line cultures and was able to attenuate T lymphocyte proliferation and function, conveying a negative regulatory role in cellular immunity [105]. In multivariate analysis, baseline plasma sPD-L1 was found to be an independent negative prognostic factor in patients with breast cancer. Similar results were reported in another study involving 222 patients with metastatic melanoma [106]. In patients with glioma, baseline circulating sPD-L1 levels were correlated with tumor grade, IDH-1 mutation status and Ki67 levels, resulting in significantly shorter OS [107]. Moreover, radiation therapy increased the mean sPD-L1 levels in those patients.

## 8. Conclusions

Apart from its well-established role in tumor immune evasion, multiple lines of evidence suggest that PD-L1 has several tumor-intrinsic properties that drive tumor progression and metastasis. Pathways linking PD-L1 expression with EMT, cancer stemness and oncogenic intracellular signaling are now in the spotlight of preclinical research. Several studies have revealed a prognostic role for PD-L1 in patients with malignancy. As data mature, more clinical trials assessing PD-1/PD-L1 axis inhibitors in early-stage cancer patients and the neoadjuvant or adjuvant treatment setting are expected to launch, allowing cancer patients to access immunotherapy early in the course of their disease.

## Figures and Tables

**Table 1:** Main pathways implicated in programmed death-ligand 1 mediated tumor progression and metastasis.

Cell Type	Pathway	Effect on PD-L1	Effect on Tumor
Cancer stem-like cells	EMT/β-catenin/STT3	N-glycosylation, stabilization	Cancer stemness, tumorigenicity
Tumor cells	PD-L1-containing exosomes	Engagement of PD-1-positive T cells in the TME, delivery of PD-L1 to different cell types in the TME	Tumor growth, metastasis
Tumor cells	KRAS, TP53/ARF6/AMAP1	Recycling	Tumor growth, fibrosis
Tumor cells	CMTM6/Wnt/β-catenin	Reduction in protein ubiquitination and lysosomal degradation	Cancer stemness, tumorigenicity, tumor growth, EMT
Tumor cells	EpEX/EGFR/ERK; MAPK	Upregulation; N-glycosylation, stabilization	Tumor growth
Tumor cells	miR-200/ZEB1	Transcriptional upregulation	EMT
Tumor cells	KRAS, MYC/eIF2a/uORF	Translational upregulation	Tumor growth, metastasis

PD-L1, programmed death-ligand 1; EMT, epithelial-to-mesenchymal transition; PD-1, programmed cell death protein 1; TME, tumor microenvironment; CMTM6, CKLF chemokine-like factor-like MARVEL transmembrane domain-containing family member 6; EpEX, EpCAM extracellular domain; EGFR, epidermal growth factor receptor; uORF, upstream open reading frame.

## Data Availability

Not applicable.
